# Predicting recovery in Bell’s palsy: the impact of early rehabilitation and prognostic indicators

**DOI:** 10.1007/s00405-025-09937-1

**Published:** 2026-01-27

**Authors:** Daniela Lucidi, Virginia Dallari, Federico Spagnolo, Giannicola Iannella, Federica Nizzoli, Elena Reggiani, Ignacio Javier Fernandez

**Affiliations:** 1 Department of Otolaryngology Head and Neck Surgery, Santa Maria Delle Croci Hospital, AUSL Della Romagna, Ravenna, Italy; 2 Alma Mater Studiorum-Università di Bologna, Bologna, Italy; 3 Young Confederation of European ORL-HNS, Y-CEORL- HNS, Vienna, Austria; 4 Department of Otolaryngology, Sassuolo Hospital, AUSL Modena, Sassuolo, Italy; 5 Department of ’Organi di Senso’, University “Sapienza”, Rome, 00185 Italy; 6 Department of Otolaryngology, University Hospital of Modena, Modena, Italy

**Keywords:** Bell’s palsy, Facial nerve, Comorbidities, Glucocorticoids, Neuromuscular retraining, Rehabilitation

## Abstract

**Purpose:**

To evaluate functional outcomes and prognostic factors in Bell’s palsy (BP) patients treated with medical therapy and facial neuromuscular retraining (fNMR), with a focus on the timing of rehabilitation.

**Methods:**

Ninety-four patients with FP treated at our center were retrospectively analyzed. Patients were stratified by time to rehabilitation (TTR): Early Rehabilitation (≤ 60 days), who accessed our unit directly, and Late Rehabilitation (> 60 days), who had first received other treatments or rehabilitation not including fNMR elsewhere. Outcomes included House–Brackmann (HB) grade, Sunnybrook score, synkinesis, and time to recovery onset (TTRO). Complete recovery was defined as HB I at 12 months.

**Results:**

At 12 months, 60 patients (63.8%) achieved complete recovery, while 34 (36.2%) did not. Median TTR was 23 days [IQR 16–68], and median TTRO was 48 days [30–113]. Early Rehabilitation was associated with significantly better outcomes at 6 and 12 months, with higher Sunnybrook scores and lower HB grades (all *p* < 0.001), and with faster TTRO.

In univariable analyses, cardiovascular and metabolic comorbidities were associated with poorer recovery. Longer TTR, delayed TTRO, higher baseline HB, lower baseline Sunnybrook, synkinesis, and previous rehabilitation were predictors of worse outcomes. At multivariable logistic regression, TTRO emerged as the only independent predictor of complete recovery (*p* = 0.017).

**Conclusions:**

Early initiation of standardized fNMR was associated with more favorable functional recovery, while delayed rehabilitation and systemic comorbidities predicted worse outcomes. Recovery beyond 12 months was exceptional, confirming the first year as the critical window for meaningful nerve regeneration.

## Introduction

Bell’s palsy (BP) is an idiopathic acute peripheral facial neuropathy causing unilateral, or less commonly bilateral, facial paralysis (FP), with an annual incidence of 20–30 cases per 100,000 people [1]. It occurs less frequently in children under 10 and the elderly, peaking between 15 and 45 years [2]. Risk factors include diabetes, pregnancy, hypertension, immunosuppression, influenza A, and upper respiratory infections [3]. The etiology is multifactorial: infectious, immune, and ischemic, with the viral hypothesis most supported [1, 4]. Pathophysiology involves nerve edema and mechanical facial nerve (FN) compression [3]. Glucocorticoids are the first-line treatment, shown to significantly improve recovery [5]. Antivirals alone are minimally effective [6], though their combination with corticosteroids is recommended in severe cases treated within 72 h [7]. While physical therapy is widely used for FN rehabilitation, its independent efficacy remains unclear. Recent evidence supports that multidisciplinary FN units improve outcomes by integrating medical, surgical, and rehabilitative care, addressing both functional and psychosocial aspects [8]. Facial neuromuscular retraining (fNMR), especially within such units, enhances recovery and reduces maladaptive movements like synkinesis [9, 10]. Most BP cases (> 70%) resolve completely within 6 months, but incomplete recovery may cause notable functional, aesthetic, and psychological impacts [7]. While early rehabilitation (within 4–6 months) is beneficial, evidence on late rehabilitation is limited, though some studies report improvements even years later in select cases [11, 12]. Assessing prognosis is essential for guiding treatment and counseling. Clinical parameters like age, comorbidities, BMI, smoking, and initial FP severity have been studied, but literature remains inconclusive regarding their prognostic value.

The primary aim of this study was to investigate the outcomes of a cohort of BP patients uniformly treated with medical therapy and fNMR at a tertiary referral institution equipped with a multidisciplinary FN unit and a dedicated rehabilitation service. Furthermore, we sought to identify clinical and therapeutic factors independently associated with FN recovery. Finally, we specifically assessed the impact of early physical rehabilitation on functional outcomes, with the ultimate goal of optimizing management strategies and guiding treatment decisions.

## Materials and methods

### Study design and population

This retrospective cohort study was conducted at the Department of Otorhinolaryngology, Head and Neck Surgery of a tertiary referral center.

#### Inclusion criteria

We assembled a consecutive cohort of adults (≥ 18 years) with acute idiopathic peripheral FP evaluated at our tertiary FN unit between January 2020 and August 2024. Eligibility required a documented symptom onset date, a first standardized assessment at our unit, including comprehensive otorhinolaryngological examination, cranial nerve function assessment, and audiometry with stapedial reflex testing, with House–Brackmann (HB) grade [13] and Sunnybrook Facial Grading System score [14] recorded, and at least 12 months of follow-up to ascertain the primary endpoint.

Patients initially managed at other facilities were eligible; for these, the onset date, baseline severity, and prior medical/rehabilitative treatments were abstracted from referral documentation and recorded for analysis.

#### Exclusion criteria

We excluded secondary FP (e.g., Lyme disease, otitis media/mastoiditis or cholesteatoma with FN involvement, temporal bone fracture, neoplasms, central causes including stroke, congenital or iatrogenic palsy), recurrent BP episodes, unknown onset date or missing baseline severity at the first assessment, and follow-up < 12 months.

BP was diagnosed by exclusion, in accordance with the clinical practice guidelines of the American Academy of Otolaryngology–Head and Neck Surgery Foundation (AAO-HNSF), which define it as a diagnosis to be made when no other identifiable medical cause for acute facial weakness is present and symptoms onset within 72 h [6, 15].

The medical treatment regimen primarily consisted of oral corticosteroids as first-line therapy, often supplemented with B-complex vitamins [16]. According to AAO-HNSF [15] and SFORL guidelines [7], antiviral therapy was administered in addition to corticosteroids in patients with severe BP (HB grades V–VI) and early symptom onset (within 72 h), or in cases of Ramsay Hunt syndrome.

FN rehabilitation was initiated within 60 days in all patients who accessed our institution early after FN palsy onset, with the exception of two cases who, despite early referral, started later due to poor adherence to follow-up.

The rehabilitation program included fNMR, facial massages (both external and intraoral techniques), and thermotherapy.

In the initial sessions, an expert speech-language pathologist instructed patients in selective motor control techniques, emphasizing slow and symmetrical contractions supported by mirror feedback to enhance neural adaptation. Patients were encouraged to perform daily home exercises to reinforce training. Massage and thermotherapy were used to reduce muscle hyperactivity, prevent post-paretic syndrome, and improve fine facial coordination.

Patients who did not show complete recovery at 15 days assessment were evaluated by a multidisciplinary team comprising an otorhinolaryngologist, neurologist, and speech therapist (FN unit). In those cases, electroneurography (ENoG), electromyography (EMG), comprehensive hematic tests, and contrast enhanced brain magnetic resonance imaging (MRI) were performed.

Referral pathways were heterogeneous: some patients accessed our service directly after onset, whereas others were referred after having been initially treated at other facilities. For referred patients, the baseline clinical parameters recorded at our institution (Sunnybrook score, HB grade, prior therapies, and ancillary tests—blood work, EMG, MRI, or ENoG when available) refer to the first evaluation at our center and may not coincide with the time of FP onset.

The treatment and observation duration was 12 months, with an extension to 24 months in cases of incomplete recovery. The progression of paralysis was assessed using the HB scale, the Sunnybrook score and synkinesis presence at follow-up visits conducted at 6 months (T6), 12 months (T12), 18 months (T18), and 24 months (T24).

Synkinesis was evaluated using the Sunnybrook Facial Grading System [14] synkinesis subscore during standardized voluntary movements. Presence of synkinesis was defined as any synkinesis score > 0 on Sunnybrook score.

For all comparative analyses, patients were classified by the time from symptom onset to initiation of fNMR, therefore referred to as time to rehabilitation (TTR), into Early Rehabilitation (≤ 60 days) and Late Rehabilitation (> 60 days). The 60-day threshold was chosen a priori to capture an early (~ 8-week) window used across prior studies (e.g., very early physical therapy within 7–10 days; trials allowing initiation up to ~ 2 months) and to precede typical synkinesis onset (~ 4–6 months) [17–20].

TTR was measured in days as the interval between the date of onset and the first fNMR session at our unit. TTR was defined as the interval between BP onset and the first fNMR session at our unit, irrespective of referral pathway or any rehabilitation previously received elsewhere.

In light of these premises, patients were further stratified into two groups based on the 12-month assessment of FN function: the Recovery Group, comprising individuals who achieved complete FN recovery (complete recovery, HB grade I at 12 months), and the No Recovery Group (HB > I), including those who did not attain full recovery.

### Data collection and outcome measures

At baseline we recorded demographics (age, sex), medical history and comorbidities (diabetes, hypertension, cardiovascular disease, obesity, migraine, smoking status, autoimmune disease), prior BP episodes, pregnancy, previous facial rehabilitation, and pharmacotherapy (corticosteroids, antivirals, B-complex vitamins). Timing variables included time to recovery onset (TTRO), defined as the days from BP onset to the first clinical signs of improvement, and TTR, as defined above. Recovery status at 12 months was classified as complete (HB grade I, Recovery Group) or incomplete (HB > I, No Recovery Group).

### Statistical analysis

Analyses were performed in SPSS v19 (IBM).

Continuous and ordinal variables (Sunnybrook score, HB grade) were summarized as mean ± standard deviation (SD) and median [interquartile range, IQR], whereas categorical variables were reported as n (%). Skewed variables (TTR/TTRO) reported as median [IQR]; group comparisons with Mann–Whitney U. Correlations were assessed with Pearson’s coefficient. Predictors of complete recovery (HB I at 12 months) were evaluated by multivariable logistic regression, while predictors of ΔSunnybrook were analyzed with linear regression. Statistical significance was set at *p* < 0.05.

## Results

### Overall population

A total of 92 patients met the inclusion criteria for unilateral FP, with one additional patient presenting with bilateral involvement. Given that the two episodes of bilateral palsy occurred 16 months apart, they were considered as separate episodes, bringing the total to 94 cases of idiopathic FP. General features of the population are summarized in Table 1.

**Table 1:** Baseline demographic characteristics of the patient cohort (cases *n* = 94, patients *n* = 92)

Characteristics	Total (*n*-%)
Patients	92
Cases of FP	94 (1 bilateral FP)
Mean age ± SD (range)	55.0 ± 20.3 years (10 − 98 years)
Mean age at symptom onset ± SD (range)	47 ± 20.8 years (3–92 years)
Gender	
Male Female	46 (48.9%) 48 (52.1%)
Side	
Left Right	50 (53.2%) 44 (46.8%)
Comorbidities	
Cardiovascular events Metabolic syndrome Diabetes mellitus Smoking Autoimmune diseases BMI > 30 Chronic headache	22 (23.4%) 10 (10.6%) 7 (7.4%) 5 (5.3%) 3 (3.1%) 1 (1.1%) 0 (0.0%)
Pregnancy	5 (5.3%)
Previous FP	7 (7.4%)
Previous rehabilitation	20 (21.3%)
No medical therapy Not available Medical therapy Corticosteroids Antiviral therapy B-complex vitamins	12 (12.8%) 7 (7.4%) 75 (79.8%) 71 (75.5%) 12 (12.8%) 56 (59.6%)
Audiometric test availability	47 (50%)
Stapedial reflex	
Present Absent	25 (53.2%) 22 (46.8%).
Blood tests availability	12 (12.8%)
Significant laboratory results	
EBV IgG positive Neutrophilic leukocytosis Elevated IgE Hyperbilirubinemia Positive serology for *Treponema pallidum* HSV-1 DNA positive (PCR)	2 (16.7%) 1 (8.3%) 1 (8.3%) 1 (8.3%) 1 (8.3%) 1 (8.3%)
Imaging performed Brain CT Brain MRI	35 (37.2%) 5 (14.3%) 30 (85.7%)
EMG performed Complete denervation Reduced CMAP amplitude Signs of reinnervation	9 (9.6%) 3 (33.3%) 4 (44.4%) 2 (22.2%)

The mean age at symptom onset was 47 ± 20.8 years (range: 3–92). Paralysis was more frequent on the left side (53.2%), with 50 cases compared to 44 cases (46.8%) on the right. The gender distribution was balanced, with 46 males (48.9%) and 48 females (51.1%), yielding an M: F ratio of 1:1.04.

Comorbidities were present in 39 patients (41.5%), of whom 7 (7.4%) had multiple conditions. The remaining 55 patients (58.5%) had no comorbidities.

As far as medical therapy is concerned, 71 (75.5%) patients received oral corticosteroids at FP onset, in line with clinical practice guidelines. Antiviral agents were prescribed in a minority of cases (12 patients, 12.8%), typically in the presence of severe BP (HB grades V–VI) and early symptom onset, or when Ramsay Hunt syndrome was suspected, in accordance with AAO-HNSF and SFORL recommendations [7, 15]. B-complex vitamins were administered in 56 patients (59.6%) based on individual physician preference and perceived potential benefit in promoting nerve regeneration [16], although their use is not universally standardized in clinical guidelines.

The average air conduction pure-tone audiometry (AC-PTA) was 20.6 ± 20.4 dB, while the bone conduction pure-tone audiometry (BC-PTA) averaged 19.6 ± 16.7 dB. The stapedial reflex was present in 54.2% of cases (25 cases), based on the available audiometric tests (47–50.0%).

Additional diagnostic investigations, such as blood tests, imaging, and EMG, are summarized in Table 1.

Among the 94 patients, 71 (75.5%) accessed our unit directly after BP onset, whereas 23 (24.5%) had initially been treated elsewhere. Rehabilitation data were available for 16 (69.6%) of these patients: 3 (18.7%) had physiotherapy, 11 (68.7%) active exercises, 1 (6.2%) Kabat therapy, and 1 (6.2%) electrostimulation. In addition, 5 (31.3%) of them had received botulinum toxin.

The temporal evolution of FP metrics, HB grade, Sunnybrook score and synkinesis grade, at baseline and follow-up time points (T0, T6, T12, T18, T24) is summarized in Table 2. These descriptives illustrate the largest gains within the first year.

**Table 2:** Summary of FP progression over time based on HB grading, Sunnybrook score and grade of synkinesis, recorded at baseline (T0), 6 months (T6), 12 months (T12), 18 months (T18) and 24 months (T24)

Parameter	T0	T6	T12	T18	T24
HB grade					
Mean ± SD Median (range)	3.7 ± 1.1 4 (2–6)	1.7 ± 0.9 1 (1–4)	1.5 ± 0.8 1 (1–4)	1.7 ± 0.9 1 (1–3)	1.8 ± 0.9 1 (1–3)
Sunnybrook score					
Mean ± SD Median (range)	47.9 ± 23.3 49 (8 -100)	89.3 ± 13.2 95 (52–100)	91.3 ± 11.9 96 (48–100)	88.5 ± 12.4 94 (65–100)	86.8 ± 12.2 86 (67–100)
Synkinesis					
Mean ± SD Median (range)	1.1 ± 2.7 0 (0–11)	2.3 ± 3.0 1 (0–17)	2.9 ± 2.8 3 (0–11)	4.8 ± 3.0 5 (0–10)	6.4 ± 1.2 6 (5–8)

**Fig. 1:**
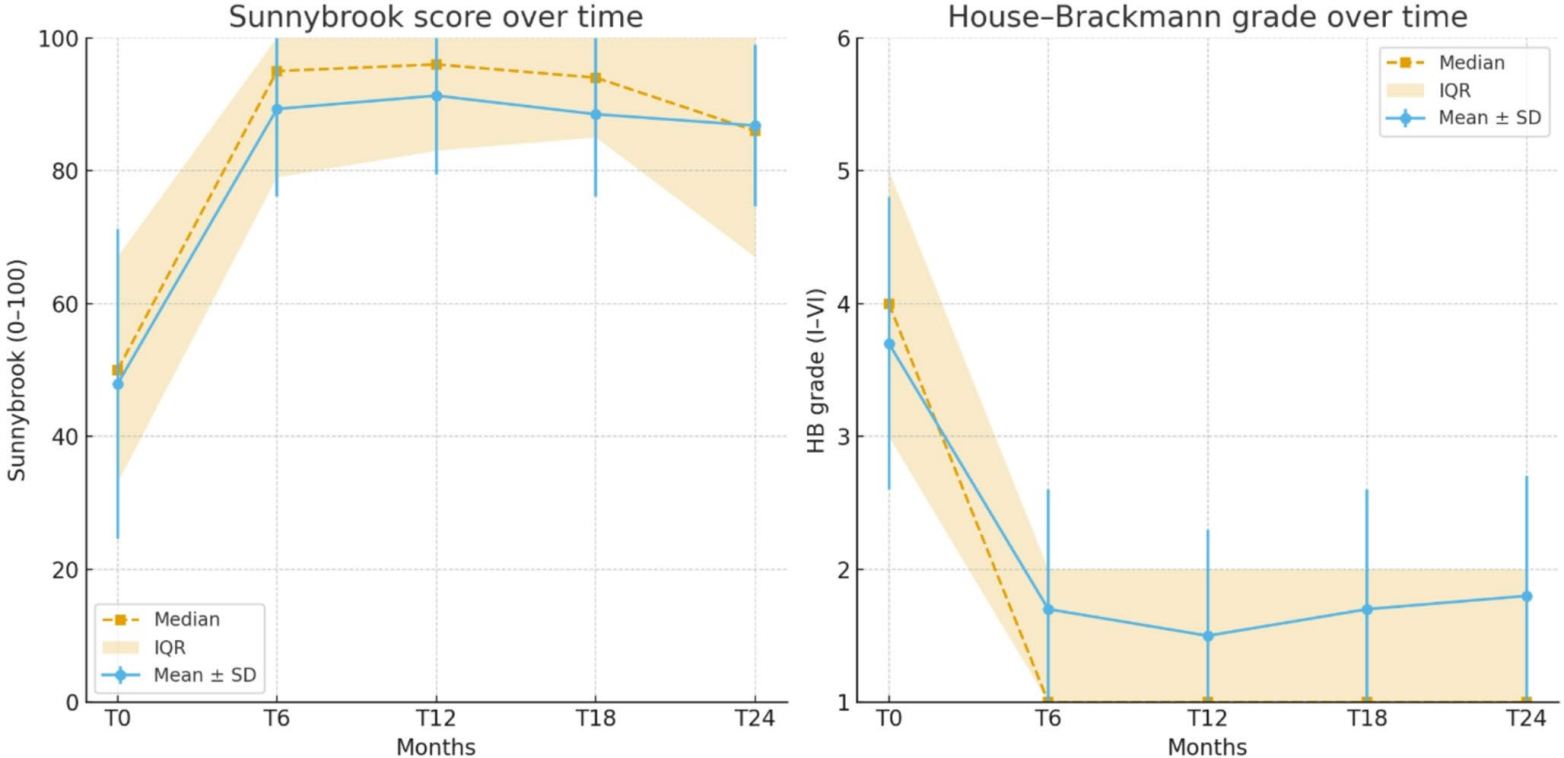
(**A**) *Sunnybrook score* over time (0–100 scale) and (**B**) *House–Brackmann grade* over time (I–VI scale). For each time point (T0, 6, 12, 18, 24 months), medians (dashed lines), interquartile range (IQR, shaded area), and mean ± SD (error bars) are shown. Sunnybrook, Sunnybrook score (0–100; higher is better). HB, House–Brackmann grade (I–VI; lower is better)

As shown in Fig. 1, both Sunnybrook and House–Brackmann scores improved markedly within the first six months, after which the curves plateaued with only minimal changes up to 24 months.

### Speech therapy and rehabilitation

Overall, patients began rehabilitation 23 days after FP onset (TTR; IQR 16–68).

The first clinical signs of recovery (TTRO) were observed at 48 days (IQR 30–113). Recovery onset occurred within 30 days in 23 patients (24.5%) and within three months in 58 patients (61.7%).

Patients were stratified into Early Rehabilitation (≤ 60 days; *n* = 69, 73.4%) and Late Rehabilitation (> 60 days; *n* = 25, 26.6%) groups. In our cohort, the Early Rehabilitation group essentially coincided with patients who accessed our unit directly after onset; the only exceptions were two patients who, despite direct access, initiated therapy after > 60 days and were therefore classified as Late due to poor follow-up adherence.

In the Early Rehabilitation group, the median TTR was 18 days (IQR 13–24) and the median TTRO was 41 days (IQR 26–60); in the Late Rehabilitation group, the median TTR was 202 days (IQR 112–623) and the median TTRO was 172 days (IQR 103–630).

All patients received a standardized program consisting of external and intraoral facial massages, thermotherapy, and fNMR.

The absolute improvement (Δ) in Sunnybrook score from baseline was 42 ± 23 (median 41, IQR 26–59) at 6 months, 43 ± 23 (median 43, IQR 27–63) at 12 months, 45 ± 22 (median 48, IQR 30–61) at 18 months, and 45 ± 24 (median 50.5, IQR 32–62) at 24 months.

The Δ in HB grade from baseline was 2.0 ± 1.4 (median 2, IQR 1–3) at 6 months, 2.2 ± 1.3 (median 2, IQR 1–3) at 12 months, 2.2 ± 1.1 (median 2, IQR 2–3) at 18 months, and 2.2 ± 1.3 (median 2, IQR 1–3) at 24 months.

**Fig. 2:**
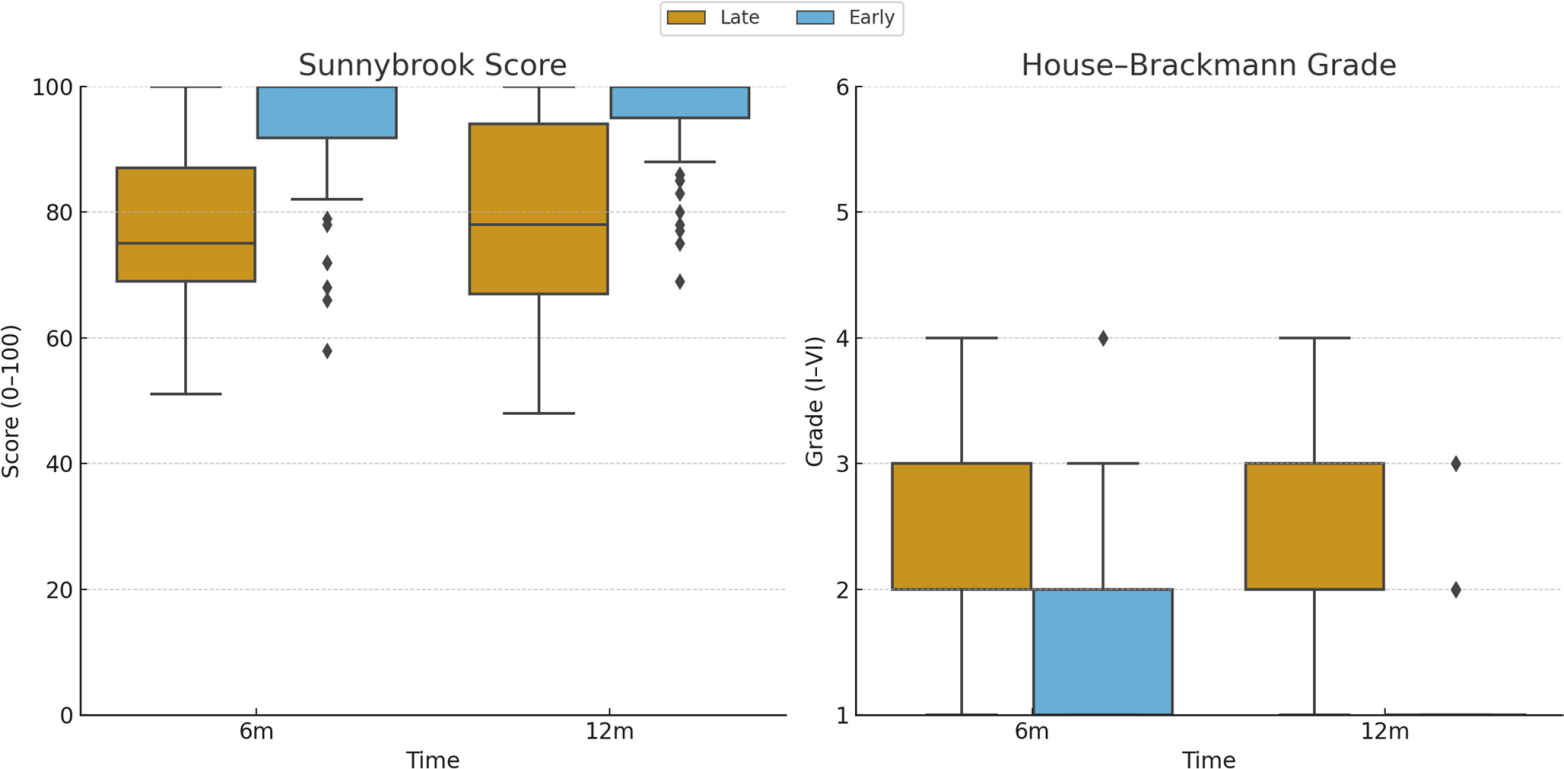
Sunnybrook score (left) and House–Brackmann grade (right) at 6 and 12 months in Early (≤ 60 days) and Late (> 60 days) Rehabilitation groups. Boxes show medians and interquartile ranges; whiskers indicate ranges; points represent individual patients. Sunnybrook score: 0–100; higher is better. House–Brackmann grade: I–VI; lower is better

At 6 months, patients in the Early Rehabilitation group showed significantly better outcomes compared with those in the Late group, with higher Sunnybrook scores (Early: mean 94.1 ± 9.5, median 100 [IQR 92–100]; Late: mean 76.2 ± 13.6, median 75 [69–87]) and lower HB grades (Early: mean 1.4 ± 0.7, median 1 [1–2]; Late: mean 2.7 ± 0.8, median 3 [2–3]). At 12 months, the difference persisted, with Early patients maintaining higher Sunnybrook scores (mean 95.5 ± 7.2, median 100 [95–100]) compared with the Late group (mean 79.6 ± 14.8, median 78 [67–94]), and lower HB grades (Early: mean 1.3 ± 0.5, median 1 [1–1]; Late: mean 2.3 ± 0.9, median 2 [2–3]) (Fig. 2).

All primary outcomes differed significantly between groups, with Early Rehabilitation associated with faster TTRO, lower HB grade, and higher Sunnybrook scores at 6 and 12 months, as well as a greater rate of complete recovery. All comparisons remained statistically significant (Mann–Whitney U test, *p* < 0.001).

All statistically significant correlations at Pearson’s analysis are displayed in Table 3.

**Table 3:** Pearson’s correlation matrix among time to recovery onset (TTRO), time to rehabilitation (TTR), and changes in Sunnybrook score (ΔT0–T6, ΔT0–T12, ΔT0–T18, ΔT0–T24)

Variable	Correlated variables	Pearson correlation	Sig. (*p*)
TTRO	TTR ΔSunnybrook T0-T6 ΔSunnybrook T0-T12 ΔSunnybrook T0-T18	0.75 -0.28 -0.26 -0.63	< 0.001 0.009 0.015 0.002
ΔSunnybrook T0-T6	TTRO TTR ΔSunnybrook T0-T12 ΔSunnybrook T0-T18 ΔSunnybrook T0-T24	-0.28 -0.22 0.97 0.93 0.94	0.009 0.035 < 0.001 < 0.001 < 0.001
ΔSunnybrook T0-T12	TTRO ΔSunnybrook T0-T6 ΔSunnybrook T0-T18 ΔSunnybrook T0-T24	-0.26 0.98 0.97 0.93	0.015 < 0.001 < 0.001 < 0.001
ΔSunnybrook T0-T18	TTRO ΔSunnybrook T0- T6 ΔSunnybrook T0-T12 ΔSunnybrook T0-T24	-0.63 0.93 0.97 0.99	0.002 < 0.001 < 0.001 < 0.001
ΔSunnybrook T0-T24	ΔSunnybrook T0-T6 ΔSunnybrook T0-T12 ΔSunnybrook T0-T18	0.94 0.93 0.99	< 0.001 < 0.001 < 0.001

TTRO was positively correlated with TTR (*r* = 0.75, *p* < 0.001), indicating that later rehabilitation was associated with a delayed recovery onset. Moreover, ΔSunnybrookT0–T6 was negatively correlated with TTR (*r*=–0.22, *p* = 0.035), suggesting that patients who started rehabilitation later showed a smaller early functional gain; this association was confirmed by linear regression analysis (*p* = 0.028).

### Recovery patients versus no recovery patients

Among 94 patients, 60 (63.8%) achieved complete recovery (HB I) at 12 months (Recovery group), whereas 34 (36.2%) did not (No Recovery group). Compared with the No Recovery group, the Recovery group was younger at onset, had shorter TTR and TTRO, and higher baseline Sunnybrook scores (Table 4). When recovery was defined more broadly as HB I–II, the 12-month recovery proportion increased to 79/94 (84.0%). Stratified by rehabilitation timing, the Early (≤ 60 days) group showed 54/69 (78.3%) for HB I and 66/69 (95.7%) for HB I–II, while the Late (> 60 days) group showed 6/25 (24.0%) for HB I and 13/25 (52.0%) for HB I–II.

**Table 4:** Baseline characteristics and follow-up by 12-month recovery status (HB I = complete recovery)

Characteristic	Recovery at 12 months	No recovery at 12 months
N	60 (63.8%)	34 (36.2%)
Age at onset, years (median [IQR])	44 [29–60]	59 [42–66]
Male sex, n (%)	31 (52%)	15 (44%)
TTR, days (median [IQR])	18 [13–24]	78 [30–260]
TTRO, days (median [IQR])	38 [24–50]	133 [80–274]
Sunnybrook at T0 (median [IQR])	52 [33–69]	38 [21–59]
Evaluated at 18 months, n	13	12
Evaluated at 24 months, n	6	6
Recovered (HB I) at 24 months among No-recovery-at-12 m, n/N (%)	—	0/6 (0%)

At follow-up, 25 patients were evaluated at 18 months (13 Recovery and 12 No Recovery) and 12 at 24 months (6 and 6, respectively). Notably, although a few recovered patients continued follow-up beyond 12 months, the majority were discharged after achieving HB I. Importantly, none of the 6 patients from the No Recovery group still under follow-up at 24 months achieved complete recovery (0/6, 0%).

At univariate analysis, several variables were significantly associated with recovery at 12 months. Patients without recovery had a higher prevalence of comorbidities, particularly cardiovascular disease and diabetes, and were more frequently exposed to previous rehabilitation not including fNMR before referral. In addition, longer TTRO, higher baseline HB grade, lower Sunnybrook scores, and the presence of synkinesis were all significantly related to poorer outcomes. Detailed results of the univariate analysis are shown in Table 5.

**Table 5:** Clinical characteristics with significant differences at univariate analysis, including comorbidities, diabetes, previous rehabilitation, time to recovery onset (TTRO), time to rehabilitation (TTR), baseline HB grade and Sunnybrook score

Variable	Complete recovery		*P*
	yes	no	
Age (years)	mean 44.2	Mean 51.6	0.085
Gender			0.42
Male	22 (47.8)	24 (52.2)	
Female	25 (52.1)	23 (47.9)	
Side			0.27
Right	24 (54.5)	20 (45.5)	
Left	23 (46.0)	27 (54.0)	
Comorbidities	32 (58.2)	23 (41.8)	0.046*
Cardiovascular events	7 (31.8)	15 (68.2)	0.043*
Metabolic syndrome	4 (40.0)	6 (60.0)	0.37
Diabetes mellitus	1 (14.3)	6 (85.7)	0.049*
Smoking	2 (60.0)	3 (40.0)	
Autoimmune diseases	1 (66.7)	2 (33.3)	
BMI > 30	0	1	0.50
Chronic headache	-	-	
Pregnancy	2 (40.0)	3 (60.0)	0.46
Previous FP	3 (42.9)	4 (57.1)	0.69
Previous rehabilitation	1 (5.0)	19 (95.0)	< 0.001*
No medical therapy	5 (41.7)	7 (58.3)	0.37
Medical therapy			
Corticosteroids	38 (53.5)	33 (46.5)	0.16
Vitamins (B6, B12)	31 (55.4)	25 (44.6)	0.14
Antiviral	5 (41.7)	7 (58.3)	0.37
Stapedial Reflex			0.19
Present	15 (62.5)	9 (37.5)	
Absent	9 (45.0)	11 (55.0)	
TTRO (days)	Mean 44.0	Mean 225.9	< 0.0001*
TTR	Mean 21.2	Mean 632.1	0.14
HB grade T0	Mean 3.5	Mean 4.1	0.038*
Sunnybrook score T0	Mean 53.4	Mean 39.6	0.005*

In multivariable analysis, TTRO was the only clinical variable independently associated with recovery (p = 0.017; Table 6). Because synkinesis grading was available for only a small subset of patients, synkinesis was excluded from the primary model to avoid quasi-separation; synkinesis outcomes are reported descriptively (Table 2). In addition, because individual comorbidities had very low counts, they are presented only in univariable analyses, whereas the multivariable model includes a single comorbidity indicator to reduce sparse-data bias. 

**Table 6:** Multivariable logistic regression analysis.

Variable	*p*-value	OR	95% CI
Comorbidities	0.21	5.06	0.41–62.2
TTRO	0.017*	0.98	0.95–0.99
HB grade T0	0.63	0.62	0.089–4.36
Sunnybrook score T0	0.50	1.033	0.94–1.13

## Discussion

BP is the most frequent cause of acute peripheral FN paralysis, though its etiology remains partly unclear, with viral reactivation of HSV-1 and VZV most strongly implicated [6]. The natural history of BP is usually favorable, with 70–80% of patients achieving full recovery, most within 6–12 months [7, 17]. However, a substantial proportion experiences persistent deficits, including synkinesis, contracture, or disfigurement, with significant psychosocial impact. Understanding prognostic factors and optimizing rehabilitation strategies is therefore essential.

In our cohort, 63.8% of patients achieved complete recovery (HB I) at 12 months, with no further significant improvement beyond this time point. This rate is slightly lower than large population-based reports [1, 21–23] yet consistent with Nakano’s review [17], which underscores heterogeneity driven by baseline severity, comorbidities, and referral patterns. The lower overall rate in our series is plausibly explained by the inclusion of late referrals, many of whom had already undergone non-fNMR rehabilitation and likely represent non-responders. Notably, outcomes among patients who accessed our unit early and initiated rehabilitation promptly were comparable to the most favorable series [23, 24]. Importantly, our outcomes may appear less favorable because we defined recovery strictly as complete normalization (HB I), rather than including partial recovery (HB ≤ II) [17, 18].

Because HB II denotes near-normal function with subtle asymmetry, we also examined a patient-centered endpoint of favorable recovery (HB I–II). Under this broader definition, the 12-month recovery rate increased from 63.8% (HB I only) to 84.0%.

Our standardized rehabilitation protocol, combining fNMR, facial massage (external and intraoral), and thermotherapy in a multidisciplinary FN unit, proved effective and reproducible. This structured approach contrasts with the heterogeneity of rehabilitation protocols reported in the literature. Physical therapy is commonly used in BP cases that persist beyond the first few months, aiming to accelerate recovery and reduce sequelae. A meta-analysis by Teixeira, which included 12 studies involving 872 patients, highlighted a high risk of bias due to differences in techniques and timing [25]. More recently, active fNMR has gained increasing support, with evidence suggesting superiority over passive modalities [17, 23]. Even in chronic FP, fNMR has shown significant benefits, as demonstrated by Karp [11], supporting its role across different stages of disease. Our findings reinforce this perspective, supporting the role of structured and active rehabilitation programs.

The most relevant finding of this study is the pivotal role of rehabilitation timing. Patients who initiated fNMR within 60 days (Early Rehabilitation) achieved significantly better outcomes than those who started later, with improved HB grade and Sunnybrook scores at both 6 and 12 months, faster recovery onset (TTRO), and higher rates of complete recovery. All between-group differences were statistically significant (Mann–Whitney U, *p* < 0.001).

Our findings are consistent with an association between earlier rehabilitation and more favorable outcomes, but the retrospective, non-randomized design precludes causal inference.

In line with prior reports, earlier retraining is linked to fewer long-term sequelae, including synkinesis [17, 23]. Urban [24] likewise found that postponing steroid therapy markedly worsened recovery, underscoring the value of prompt management. Kim et al. [20] showed that beginning treatment within 3 months significantly lowered long-term synkinesis, while our data indicate that starting rehabilitation within 60 days correlates with greater functional gains over time. Although these studies target different outcomes, both support timely rehabilitation to optimize results and limit persistent deficits.

In our cohort, Early Rehabilitation was defined as initiation within 60 days of onset; nevertheless, patients in this group actually began therapy at a median of 18 days (IQR 13–24). This pattern suggests that, while a 60-day cut-off is clinically meaningful, earlier initiation further increases the likelihood of optimal recovery.

Pearson’s correlation analysis confirmed a strong relationship between TTRO and TTR (*p* < 0.001), indicating that delayed initiation of therapy directly translated into delayed recovery onset. Moreover, TTR correlated significantly with ΔSunnybrookT0–T6 (*p* = 0.035), and this association was confirmed in a linear regression model (*p* = 0.028). At multivariable analysis, TTRO remained the only independent predictor of recovery (*p* = 0.017), underscoring the prognostic importance of early recovery dynamics. Taken together, these findings highlight that both the timing of rehabilitation and the trajectory of early recovery are crucial determinants of long-term outcome.

Comorbidities were frequent in our cohort (41.5% had ≥ 1 chronic condition). On univariable analysis, cardiovascular disease and diabetes were associated with poorer outcomes (both *p* < 0.05), consistent with prior reports [1, 23, 24]. However, these associations were not significant in the multivariable model; TTRO remained the only independent predictor. This pattern suggests residual confounding (e.g., baseline severity, referral pathways), despite the biological plausibility of a vascular–metabolic influence on nerve recovery described in the literature. The impact of systemic factors on FN recovery has also been confirmed in surgical cohorts, where age and vascular burden strongly influenced prognosis [26]. The specific association between hypertension and BP, with reports of resolution after antihypertensive therapy, further highlights the role of vascular factors in disease pathophysiology [27, 28].

In our cohort, the greatest improvement in HB grade and Sunnybrook score occurred within the first 12 months, with significant gains already evident at 6 months (Fig. 1). After this period, scores tended to stabilize, confirming that the critical window for functional recovery extends to the first year [1, 23]. Synkinesis progressively increased between 6 and 12 months, consistent with late reinnervation phenomena (Table 2). The apparent increase at 18 and 24 months should therefore be interpreted with caution, as the late sample is enriched for non-recovered patients and likely overestimates the true prevalence in the overall cohort.

Patients in the Late Rehabilitation group (> 60 days) consistently performed worse. This is not only due to the detrimental effect of delayed rehabilitation, but also reflects referral bias: these patients had already failed to recover spontaneously and often underwent previous, non-standardized rehabilitation elsewhere. Nevertheless, this reinforces the conclusion that early initiation of structured therapy maximizes recovery, while late initiation is associated with a reduced likelihood of full restitution. Recent studies confirm the value of structured rehabilitation: fNMR improves outcomes after surgical FP [29, 30], and its combination with botulinum toxin further reduces synkinesis [31].

Overall, our results demonstrate that structured rehabilitation in a specialized FN unit, initiated within 60 days, is associated with superior outcomes, with robust statistical support (Mann–Whitney U, *p* < 0.001 for HB and Sunnybrook at 6 and 12 months; Pearson *p* < 0.001 for TTRO–TTR correlation; *p* = 0.035 for ΔSunnybrookT0–T6; regression *p* = 0.028; multivariable *p* = 0.017). These findings are consistent with the literature supporting the superiority of fNMR and the decisive impact of rehabilitation timing [17, 23].

In line with recent randomized trials, structured exercise programs, including mirror-based protocols and neural mobilization, have demonstrated greater functional gains than usual care or conventional exercise in acute BP. Taken together, these trials support a role for targeted, protocolized physiotherapy, while acknowledging heterogeneity and risk of bias across studies. Consistently, our results, showing higher proportions of favorable recovery with earlier rehabilitation, align with this evolving evidence base, although our retrospective design precludes causal inference [32–34].

Emerging randomized data also suggest that telerehabilitation-based exercise therapy may achieve outcomes comparable to in-person delivery in peripheral FP, potentially improving access and adherence. Although preliminary, these findings align with our emphasis on early, structured retraining within a monitored pathway [35].

Our study has some limitations. The retrospective design carries inherent biases in patient selection and follow-up. The inclusion of late referrals reduces homogeneity and complicates direct comparisons with trial populations. Moreover, long-term data beyond 12 months may be affected by attrition bias, as patients achieving full recovery often discontinued follow-up, leaving only those with persistent deficits under observation. Finally, the absence of a control group precludes definitive conclusions on the superiority of our protocol over other modalities, and the single-center setting may limit generalizability.

Despite these limitations, the strengths of this study are notable. It represents one of the largest cohorts uniformly treated with a structured rehabilitation program in a dedicated multidisciplinary FN unit. The consistency of our results across multiple outcome measures (HB, Sunnybrook, TTRO), the demonstration of the decisive role of rehabilitation timing, and the identification of TTRO as an independent predictor provide robust real-world evidence.

Future perspectives include the integration of artificial intelligence (AI) tools into clinical practice, which may enhance the objectivity of FN assessment and support rehabilitation monitoring. Recent studies have demonstrated the potential of AI for automated grading, synkinesis detection, and functional tracking in FP [36, 37]. Moreover, the growing application of large language models in otorhinolaryngology [38] suggests that AI-driven platforms may also assist in clinical decision-making and patient education, complementing multidisciplinary care pathways.

## Conclusions

Early initiation of structured fNMR within 60 days from symptom onset, alongside appropriate medical therapy, was associated with more favorable functional recovery in patients with BP. In our cohort, the first year appeared to represent the critical window for nerve regeneration, as patients who did not achieve full recovery within 12 months showed no further improvement at 24 months. Cardiovascular and metabolic comorbidities were associated with worse outcomes, whereas TTRO emerged as the only independent predictor, underscoring the relevance of early recovery dynamics. Patients who started rehabilitation after the 60-day threshold had lower recovery rates than those treated earlier. Given the retrospective, non-randomized design, these findings should be interpreted as associations rather than causal effects. They support consideration of integrating medical therapy with structured fNMR programs within multidisciplinary FN units and underscore the rationale for early referral and adherence to rehabilitation pathways; prospective randomized or well-designed prospective cohort studies are warranted to establish causal effects and define optimal timing.

## References

[1] Psillas G, Dimas GG, Sarafidou A et al (2021) Evaluation of effects of diabetes Mellitus, hypercholesterolemia and hypertension on bell’s palsy. J Clin Med 10(11):2357. 10.3390/jcm10112357 34072018 10.3390/jcm10112357 PMC8198958

[2] Kim SH, Jung J, Jung SY et al (2019) Comparative prognosis in patients with Ramsay-Hunt syndrome and bell’s palsy. Eur Arch Otorhinolaryngol 276(4):1011–1016. 10.1007/s00405-019-05300-3 30707280 10.1007/s00405-019-05300-3

[3] Dalrymple SN, Row JH, Gazewood JD (2025) Bell’s palsy. Prim Care 52(1):111–121. 10.1016/j.pop.2024.09.012 39939082 10.1016/j.pop.2024.09.012

[4] Gagyor I, Madhok VB, Daly F, Sullivan F (2019) Antiviral treatment for bell’s palsy (idiopathic facial paralysis). Cochrane Database Syst Rev 9:CD001869. 10.1002/14651858.CD001869.pub9 31486071 10.1002/14651858.CD001869.pub9 PMC6726970

[5] Madhok VB, Gagyor I, Daly F et al (2016) Corticosteroids for bell’s palsy (idiopathic facial paralysis). Cochrane Database Syst Rev 7:CD001942. 10.1002/14651858.CD001942.pub5 27428352 10.1002/14651858.CD001942.pub5 PMC6457861

[6] Zhu Y, Liang T, Liu S et al (2025) Comparison of glucocorticoids plus antivirals vs glucocorticoids alone in bell’s palsy: meta-analysis of RCTs. Am J Otolaryngol 46(1):104583. 10.1016/j.amjoto.2024.104583 39724817 10.1016/j.amjoto.2024.104583

[7] Fieux M, Franco-Vidal V, Devic P et al (2020) SFORL guidelines: management of acute bell’s palsy. Eur Ann Otorhinolaryngol Head Neck Dis 137(6):483–488. 10.1016/j.anorl.2020.06.004 32636146 10.1016/j.anorl.2020.06.004

[8] Kimber R, Rodger A, Higgins R, Christofi G (2024) Combined facial neuromuscular rehab + reconstruction: MDT approach. Facial Plast Surg 40(4):407–417. 10.1055/s-0044-1779044 38286419 10.1055/s-0044-1779044

[9] Butler DP, Grobbelaar AO (2017) Facial palsy: what can the multidisciplinary team do? J Multidiscip Healthc 10:377–381. 10.2147/JMDH.S125574 29026314 10.2147/JMDH.S125574 PMC5626419

[10] Steinhäuser J, Volk GF, Thielker J et al (2022) Multidisciplinary care of facial palsy: 1220 patients in a German FN center. J Clin Med 11(2):427. 10.3390/jcm11020427 35054119 10.3390/jcm11020427 PMC8778429

[11] Karp E, Waselchuk E, Landis C et al (2019) Facial rehabilitation as noninvasive treatment for chronic FN paralysis. Otol Neurotol 40(2):241–245. 10.1097/MAO.0000000000002107 30624409 10.1097/MAO.0000000000002107

[12] Watson GJ, Glover S, Allen S, Irving RM (2015) Outcome of facial physiotherapy in prolonged idiopathic FP. J Laryngol Otol 129(4):348–352. 10.1017/S0022215115000675 25782592 10.1017/S0022215115000675

[13] House JW, Brackmann DE (1985) Facial nerve grading system. Otolaryngol Head Neck Surg 93(2):146–147. 10.1177/019459988509300202 3921901 10.1177/019459988509300202

[14] Pavese C, Tinelli C, Furini F et al (2013) Italian validation of Sunnybrook. Neurol Sci 34(4):457–463. 10.1007/s10072-012-1025-x 22487815 10.1007/s10072-012-1025-x

[15] Baugh RF, Basura GJ, Ishii LE et al (2013) Clinical practice guideline: bell’s palsy (AAO-HNSF). Otolaryngol Head Neck Surg 149(3 Suppl):S1–27. 10.1177/0194599813505967 24189771 10.1177/0194599813505967

[16] Ehmedah A, Nedeljkovic P, Dacic S et al (2020) Vitamin B complex and Schwann cell–macrophage association after PNI. Molecules 25(22):5426. 10.3390/molecules25225426 33228193 10.3390/molecules25225426 PMC7699497

[17] Nakano H, Fujiwara T, Tsujimoto Y et al (2024) Physical therapy for peripheral FP: systematic review & meta-analysis. Auris Nasus Larynx 51(1):154–160. 10.1016/j.anl.2023.04.007 37149416 10.1016/j.anl.2023.04.007

[18] Nicastri M, Mancini P, De Seta D et al (2013) Efficacy of early physical therapy in severe bell’s palsy: a randomized controlled trial. Neurorehabil Neural Repair 27(6):542–551. 10.1177/1545968313481280 23549520 10.1177/1545968313481280

[19] Khan AJ, Szczepura A, Palmer S et al (2022) Physical therapy for facial nerve paralysis (Bell’s palsy): an updated and extended systematic review of the evidence for facial exercise therapy. Clin Rehabil 36(11):1424–1449. 10.1177/02692155221110727 35787015 10.1177/02692155221110727 PMC9510940

[20] Kim DR, Kim JH, Jung SH et al (2023) Neuromuscular retraining for early severe bell’s palsy minimizes synkinesis. Clin Rehabil 37(11):1510–1520. 10.1177/02692155231166216 36972474 10.1177/02692155231166216

[21] Peitersen E (2002) Bell's palsy: the spontaneous course of 2,500 peripheral facial nerve palsies of different etiologies. Acta Otolaryngol Suppl (549):4–30 12482166

[22] Yeo SW, Lee DH, Jun BC, Chang KH, Park YS (2007) Prognostic factors in bell’s palsy & Ramsay Hunt. Auris Nasus Larynx 34(2):159–164. 10.1016/j.anl.2006.09.005 17055202 10.1016/j.anl.2006.09.005

[23] Yoo MC, Soh Y, Chon J et al (2020) Factors associated with favorable outcomes in adult bell palsy. JAMA Otolaryngol Head Neck Surg 146(3):256–263. 10.1001/jamaoto.2019.4312 31971554 10.1001/jamaoto.2019.4312 PMC6990801

[24] Urban E, Volk GF, Geißler K et al (2020) Prognostic factors for bells’ palsy outcome. Clin Otolaryngol 45(5):754–761. 10.1111/coa.13571 32395899 10.1111/coa.13571

[25] Teixeira LJ, Valbuza JS, Prado GF (2011) Physical therapy for Bell’s palsy (idiopathic facial paralysis). Cochrane Database Syst Rev (12):CD006283 Published 2011 Dec 7. 10.1002/14651858.CD006283.pub3 10.1002/14651858.CD006283.pub3 PMC12933289 22161401

[26] Lucidi D, Marchioni D, Bisi N et al (2025) Prognostic factors for FN outcome after VS surgery. Otol Neurotol 46(8):978–983. 10.1097/MAO.0000000000004582 40501311 10.1097/MAO.0000000000004582

[27] Ellis SL, Carter BL, Leehey MA, Conry CM (1999) Bell’s palsy with uncontrolled hypertension. Ann Pharmacother 33(12):1269–1273. 10.1345/aph.19129 10630827 10.1345/aph.19129

[28] Lavin PJ, Weissman BM (1985) Bell’s palsy’ in accelerated hypertension. Postgrad Med 77(8):165–168. 10.1080/00325481.1985.11699035 4001036 10.1080/00325481.1985.11699035

[29] Mattioli F, Galloni C, Alberti C et al (2025) Facial nerve graft in malignant tumors: role of rehab. J Clin Med 14(3):968. 10.3390/jcm14030968 39941638 10.3390/jcm14030968 PMC11818287

[30] Molinari G, Calvaruso F, Barbazza A et al (2024) Recovery patterns after nerve-sparing Parotid surgery: role of NMR. Eur Arch Otorhinolaryngol 281(10):5465–5472. 10.1007/s00405-024-08758-y 38914817 10.1007/s00405-024-08758-y PMC11416383

[31] Bonali M, Federico C, Tozzi A et al (2025) NMR + botulinum toxin A: experience & new protocol. Eur Arch Otorhinolaryngol. 10.1007/s00405-025-09465-y. online May 2241206396

[32] Martineau S, Rahal A, Piette E, Moubayed S, Marcotte K (2022) The mirror effect plus protocol for acute bell’s palsy: A randomized controlled trial with 1-year follow-up. Clin Rehabil 36(10):1292–1304. 10.1177/02692155221107090 35722671 10.1177/02692155221107090 PMC9420890

[33] Santiago S, Joshua AM, Nayak A et al (2024) Effectiveness of novel facial stretching with structured exercise versus conventional exercise for Bell’s palsy: a single-blinded randomized clinical trial. Sci Rep 14(1):13266. Published 2024 Jun 10. 10.1038/s41598-024-64046-z 10.1038/s41598-024-64046-z PMC11164989 38858464

[34] Alharbi R, Kashoo FZ, Ahmed M et al (2023) Effect of neural mobilisation in bell’s palsy: A randomised controlled trial. Hong Kong Physiother J 43(2):93–103. 10.1142/S1013702523500063 37583924 10.1142/S1013702523500063 PMC10423671

[35] Özden F, Golcuk Y, Tümtürk İ, Özkeskin M (2024) The effects of Telerehabilitation-Based exercise therapy on motor and Non-Motor clinical outcomes in adults with facial palsy: A randomized controlled trial. Percept Mot Skills 131(6):2182–2198. 10.1177/00315125241284677 39351643 10.1177/00315125241284677

[36] Gaber A, Taher MF, Wahed MA, Shalaby NM, Gaber S (2022) ML-based classification of facial paralysis. Biomed Eng Online 21(1):65. 10.1186/s12938-022-01036-0 36071434 10.1186/s12938-022-01036-0 PMC9449956

[37] Shi H, Fan Y, Zhang Y et al (2024) Intelligent bell facial paralysis assessment with improved SSD. Sci Rep 14(1):12763. 10.1038/s41598-024-63478-x 38834661 10.1038/s41598-024-63478-x PMC11150464

[38] Trecca EMC, Caponio VCA, Turri-Zanoni M et al (2025) Info quality in pediatric ORL: clinicians, residents & LLMs. Otolaryngol Head Neck Surg 173(1):228–236. 10.1002/ohn.1225 40105497 10.1002/ohn.1225 PMC12207379

